# Sitagliptin Modulates Oxidative, Nitrative and Halogenative Stress and Inflammatory Response in Rat Model of Hepatic Ischemia-Reperfusion

**DOI:** 10.3390/antiox10081168

**Published:** 2021-07-22

**Authors:** Małgorzata Trocha, Mariusz G. Fleszar, Paulina Fortuna, Łukasz Lewandowski, Kinga Gostomska-Pampuch, Tomasz Sozański, Anna Merwid-Ląd, Małgorzata Krzystek-Korpacka

**Affiliations:** 1 Department of Pharmacology, Wroclaw Medical University, 50-345 Wroclaw, Poland; tomasz.sozanski@umed.wroc.pl (T.S.); anna.merwid-lad@umed.wroc.pl (A.M.-L.); 2 Department of Biochemistry and Immunochemistry, Wroclaw Medical University, 50-368 Wroclaw, Poland; mariusz.fleszar@umed.wroc.pl (M.G.F.); paulina.fortuna@umed.wroc.pl (P.F.); lukasz.lewandowski@umed.wroc.pl (Ł.L.); kinga.gostomska-pampuch@umed.wroc.pl (K.G.-P.)

**Keywords:** drug repurposing, dipeptidylpeptidase-4 antagonists, midkine, bromotyrosine, nitrotyrosine, liver transplantation, NADPH oxidase (NOX), hepatoprotection

## Abstract

A possibility of repurposing sitagliptin, a well-established antidiabetic drug, for alleviating injury caused by ischemia-reperfusion (IR) is being researched. The aim of this study was to shed some light on the molecular background of the protective activity of sitagliptin during hepatic IR. The expression and/or concentration of inflammation and oxidative stress-involved factors have been determined in rat liver homogenates using quantitative RT-PCR and Luminex^®^ xMAP^®^ technology and markers of nitrative and halogenative stress were quantified using targeted metabolomics (LC-MS/MS). Animals (n = 36) divided into four groups were treated with sitagliptin (5 mg/kg) (S and SIR) or saline solution (C and IR), and the livers from IR and SIR were subjected to ischemia (60 min) and reperfusion (24 h). The midkine expression (by 2.2-fold) and the free 3-nitrotyrosine (by 2.5-fold) and IL-10 (by 2-fold) concentration were significantly higher and the *Nox4* expression was lower (by 9.4-fold) in the IR than the C animals. As compared to IR, the SIR animals had a lower expression of interleukin-6 (by 4.2-fold) and midkine (by 2-fold), a lower concentration of 3-nitrotyrosine (by 2.5-fold) and a higher *Nox4* (by 2.9-fold) and 3-bromotyrosine (by 1.4-fold). In conclusion, IR disturbs the oxidative, nitrative and halogenative balance and aggravates the inflammatory response in the liver, which can be attenuated by low doses of sitagliptin.

## 1. Introduction

The liver is the second most frequently transplanted organ worldwide. The transplantation procedure is the best and often the last option available for patients with end-stage liver disease. Although its frequency is steadily increasing, the number of patients awaiting transplantation vastly exceeds organ availability [1]. Liver transplantation is associated with the risk of graft rejection and ischemia/reperfusion (IR). It is estimated that the accompanying IR injury is responsible for 10% of early organ failure [1]. The pathogenesis of liver injury during IR is complex and still not fully understood. The initial damage is caused by ischemia and aggravated by reperfusion, which involves an early acute (3–6 h after reperfusion) and a subacute phase (18–24 h after reperfusion). While an acute phase is associated with Kupffer cells activation, the subacute phase is characterized by neutrophil infiltration [2,3]. Reperfusion, in each phase, is accompanied by the accelerated generation of reactive oxygen (ROS), nitrogen (RNS) and halogen (RHS) species. Their production is incited by inflammatory cues and, in turn, ROS, RNS and RHS perpetuate and exacerbate an ongoing inflammation by upregulating cytokine and chemokine release [4,5]. Halogenated and nitrated derivatives of tyrosine are used as markers of leukocyte-mediated tissue damage. Bromotyrosine (BT) indicates eosinophils’ activation as eosinophil peroxidase preferentially generates hypobromous acid, even though bromide concentrations are lower than those of chloride even by 1000-fold. Chlorotyrosine (CT) and nitrotyrosine (NT), in turn, are considered to be markers of neutrophils and monocyte/macrophage activation [5,6,7]. NT is referred to as a footprint of inducible nitric oxide synthase (NOS2) [8]. It is worth mentioning, however, that reversible protein modification by ROS, RNS and RHS has also a beneficial aspect, being involved in regulating a number of signal transduction pathways [5].

Unraveling IR mechanisms has been a research focus for over a decade as it is a prerequisite for developing effective graft protecting strategies [1]. Extending the indications of already known drugs [9] can reduce the costs and time needed for the development of new drugs, as some critical features such as toxicity, pharmacokinetics and pharmacodynamics are already known. In line with this trend, the potential of dipeptidyl peptidase-4 (DPP-4) inhibitors is investigated and discovered. These are well-known antidiabetic drugs that have recently been shown to ameliorate IR damage in organs [10,11,12,13,14]. The rationale is that DPP-4 is involved in the metabolism of many bioactive peptides that act as chemokines, hormones and neuromodulators [15,16] and DPP-4 inhibitors have been shown to display cytoprotective properties, also via DPP4-independent mechanisms [17].

Several experimental studies have demonstrated pleiotropic properties, including the antioxidative, anti-inflammatory and antiapoptotic action of sitagliptin [18,19], a well-tolerated DPP-4 inhibitor with moderate side effects [20]. Accordingly, we [21,22], as well as others [23,24,25,26,27,28,29,30,31,32,33], have shown that sitagliptin alleviates injury caused by IR procedure. However, the molecular background has not been fully elucidated. Therefore, this follow-up study was designed to shed some light on mechanism behind the beneficial properties of sitagliptin in the liver. The drug effects on the expression (*Il6*, *Tnfa*, *Mdk*, *Ptn*, *Nampt*, *Mmp1*, *Nox1*, *Nox2* and *Nox4*) and/or secretion (IL-1β, IL-6, IL-10, IL-12(p70), IL-13, IFN-γ, MIP-2, TNF-α and VEGF-A) of inflammatory mediators known to be upregulated during IR and/or downregulated by sitagliptin were evaluated. In addition, the markers of oxidative, nitrative and halogenative stress (NT, CT and BT) were examined. We found that hepatic IR injury is associated with disturbed nitrative and halogenative balance in addition to oxidative stress and inflammatory response and demonstrated sitagliptin’s ability to attenuate it by reducing the expression of inflammatory agents and modulating oxidative, nitrative and halogenative stress.

## 2. Materials and Methods

This is a follow-up study conducted on biobanked biological material collected during the original experiment [21,22]. Its design as well as the analytical methods used for the purpose of the current study are detailed below.

### 2.1. Animals

The study was carried out on 2–3-month-old male Wistar rats. Animals were kept individually at a 12:12 h light–dark cycle, humidity 45–60%, continuous ventilation at 21–23 °C. Animals had free access to standard food and water prior experiment.

### 2.2. Chemicals

The following drugs and chemicals were used: sitagliptin (Januvia—tabl. 100 mg) purchased from MSD (Warsaw, Poland), ketamine hydrochloride (Bioketan) from Vetoquinol Biowet (Gorzów Wlkp. Poland), heparin (Heparinum WZF—amp. 25,000 U/5 mL) from Polfa Warszawa (Warsaw, Poland), butorphanol tartrate (Morphasol, amp. 4 mg/mL) from aniMedica GmbH (Frankfurt am Main, Germany), medetomidine hydrochloride (Domitor, amp. 1 mg/mL) from Orion Pharma (Danderyd, Sweden), solution of 0.9% sodium chloride from Polpharma S.A. (Starogard Gdański, Poland) and Ringer solution from Polfa Lublin S.A. (Lublin, Poland).

Methanol, acetonitrile, acetone, water, formic acid (FA) and trifluoroacetic acid (TFA) were acquired from Merck Millipore (Warsaw, Poland). Standards of 3-Nitro-l-tyrosine (NT), 3-Chloro-l-tyrosine (CT) and 3-Bromo-l-tyrosine (BT) were obtained from Sigma-Aldrich (Poznan, Poland) and Toronto Research Chemicals (Toronto, ON, Canada). Isotope-labeled standards of 3-Nitro-l-tyrosine (RING-13C6, 99%; 3-NT-13C6), 3-Chloro-l-tyrosine (RING-13C6, 99%; 3-CT-13C6) and 3-Bromo-l-tyrosine (RING-13C6, 99%; 3-BT-13C6) were procured from Cambridge Isotope Laboratories (Tewksbury, MA, USA).

### 2.3. IR Procedure

After adaptation, rats were divided randomly into the following four groups: control (n = 9) and sitagliptin (n = 8), in which animals underwent a sham-operation (no IR procedure), and IR (n = 9) and SIR (n = 10), in which animals were subjected to an IR procedure. For two weeks prior to surgery, rats from sitagliptin and SIR groups were receiving sitagliptin (5 mg/kg p.o.) once a day. Sitagliptin was applied intragastrically via a gastric tube. The drug was dissolved in 0.9% NaCl and animals received 4 mL of solution per 1 kg of body weight.

Animals were anesthetized prior surgical procedure by an intramuscular injection of ketamine hydrochloride (7 mg/kg), medetomidine hydrochloride (0.1 mg/kg) and butorphanol tartrate (2 mg/kg). Subsequently, they were subjected to a midline laparotomy. In IR and SIR groups, ischemia was induced in 70% of the liver (median and left lateral lobes) by the clamping of the portal vein and hepatic artery with a microvascular clip. After 60 min, the clip was removed to allow reperfusion for 24 h. When the experiment was terminated, livers were weighted and ischemic lobes were isolated and portioned and either placed in RNAlater (Qiagen, Hilden, Germany) for transcriptomic or frozen and stored at −80 °C for cytokine profiling and metabolomic analysis.

Animals from the sitagliptin and control groups underwent surgery, in which isolated blood vessels were not occluded after laparotomy. All surgical procedures were blindly performed by the same experienced surgeons.

The presence of IR injury was confirmed by both the determination of the activity of aminotransferases during the reperfusion and the histological evaluation under a light microscope of different regions of ischemic and non-ischemic lobes of livers after IR procedure [22]. The activity of transaminases after reperfusion was the highest in the IR group. The use of sitagliptin resulted in the ALT activity in the treated groups being significantly lower compared to the untreated groups independent of the IR procedure. Histological examination revealed no significant difference in the liver structure between ischemic and non-ischemic rats. The exception was a slight degree of necrosis and neutrophil infiltration in ischemic groups IR and SIR. The animals treated with sitagliptin (sitagliptin and SIR) showed a higher percentage of steatosis than the non-treated animals (control and IR) [22].

### 2.4. Analytical Methods

#### 2.4.1. Transcriptomic Analysis

RNeasy Mini Kit from Qiagen was used for isolating total RNA from harvested livers. Potential contamination with genomic DNA was avoided by an on-column digestion with DNase using RNase-Free DNase Set (Qiagen, Hilden, Germany). Isolated RNA was quantified and tested for purity using a NanoDrop2000 spectrophotometer from Thermo Fisher Scientific (Waltham, MA, USA). Aliquots of RNA (500 ng) were reversely transcribed to cDNA according to manufacturer’s instructions using iScript (BioRad, Hercules, CA, USA).

All qPCRs were performed in triplicates using CFX96 thermocycler (Biorad, Hercules, CA, USA) and standardized thermal cycling conditions. Polymerase was activated at 95 °C for 110 s and, subsequently, 40 cycles of denaturation at 95 °C for 5 s and annealing and synthesis at 61.4 °C for 5 s were applied and followed by a melting step (60–95 °C, reading every 0.5 °C) to confirm product specificity. Reaction mixture contained 10 µL of 2× SsoFast EvaGreen^®^ Supermix (BioRad, Hercules, CA, USA), 2 µL of cDNA (diluted 1:5), 1 µL of each 10 nM forward and reverse target-specific primers and water up to 20 µL. Primers were synthesized by Genomed (Warsaw, Poland). Their sequences, as proposed by Origene (www.origene.com, assessed on 10 June 2021), are presented in Table 1. The relative expression of genes of interest was expressed as normalized relative quantities (NRQ) [34], calculated in the following manner: geometric mean of all Cq values was subtracted from individual sample Cq (ΔCq), linearized by 2^ΔCq^ conversion and normalized to *Gapdh* expression.

#### 2.4.2. Liver Homogenates and Protein Quantification

Rat livers (0.4–0.5 g) were homogenized using ceramic lysing matrix beads in the FastPrep-24™ homogenizer (MP Biochemicals, Solon, OH, USA) with two volumes of Tris-EDTA buffer pH 7.2 (10 mM Tris, 1 mM EDTA, 1 mM MgCl_2_ and 150 mM KCl) with 1 mM PMSF, 1% deoxycholate and 1% Triton X-100 Obtained homogenates were centrifuged (14,000× *g*, 10 min, 4 °C) and supernatants were collected, aliquoted and stored at −80 °C until the analyses. For the purpose of metabolic analysis, additional tissue homogenization in water instead of lysis buffer was conducted using Bead Ruptor Elite bead mill homogenizer (Omni International, Kennesaw, GA, USA).

Prior to any analysis, tissue homogenates were clarified by centrifugation at 10,000× *g* for 10 min at 4 °C.

Protein was quantified using Coomassie Plus (Bradford) Assay Kit (Thermo Fisher Scientific, Waltham, MA, USA) against the bovine serum albumin (BSA) standard curve, according to the manufacturer’s instructions. Samples were diluted 1:10 in PBS and tested in duplicates.

#### 2.4.3. Cytoprofiling

Interleukin (IL)-1β, IL-6, IL-13, IL-10, IL-12(p70), interferon (IFN)-γ, vascular endothelial growth factor (VEGF)-A, macrophage inflammatory protein (MIP)-2 and tumor necrosis factor (TNF)-α concentrations were determined using the MILLIPLEX MAP Rat Cytokine/Chemokine Magnetic Bead Panel (Merck Millipore, Darmstadt, Germany) following manufacturer’s instructions. Samples were diluted 1:50 and tested in duplicates. The analysis was performed on a MAGPIX Instrument (Merck Millipore, Darmstadt, Germany) using Xponent Software 4.2. Data were normalized to protein content and expressed as pg/mg of protein.

#### 2.4.4. Metabolomic Analysis

Free CT, BT and NT were measured on the basis of the previously developed method [35]. Briefly, 100-microliter aliquots of homogenates or calibration standards with 20 µL of 0.2% TFA and 10 µL of internal standard (isotope labeled analogs of CT, BT and NT) were mixed for one minute. After that samples were extracted with 200 µL of acetone at 25 °C for 10 min and centrifuged at 20,000× *g* for 5 min at 4 °C. The obtained supernatants were evaporated to dryness, and the residue was re-dissolved in 30 µL of 0.1% FA in water, directly before LC-MS analysis.

LC-MS data were obtained using Thermo Scientific Accela UPLC system (Thermo Scientific, Waltham, MA, USA) equipped with a triple quadrupole mass analyzer equipped with an electrospray (ESI) ion source (TSQ Quantum Access MAX Triple Quadrupole MS, Thermo Scientific, Waltham, MA, USA). Spectra were obtained in positive ionization mode with the following MS parameters: the sprayer voltage, vaporizer temperature and the capillary temperature were set at 3 kV, 265 °C and 355 °C. All scans were carried out in a selected reaction monitoring (SRM) mode. Calculations were performed with Xcalibur 2.1 software (Thermo Scientific, Waltham, MA, USA).

Analytes were separated using a Kinetex PFP chromatographic column (100 × 2.1 mm, 1.70 µm) from Phenomenex (Torrance, CA, USA) with a linear gradient from 10 to 90% of mobile phase B in 8.5 min with a total flow rate of 300 µL/min. As mobile phases, 0.1% FA in water (A) and 0.1% FA in methanol (B) were used.

Using standard lysis buffer for tissue homogenization allowed for the quantification of BT, while water-based homogenates were used for NT determination. The CT concentration was too low to be quantified, regardless of the method of homogenization applied.

### 2.5. Statistical Analysis

Data distribution and homogeneity of variances were analyzed using the Kolmogorov–Smirnov and Levene test, respectively. Raw data were log-transformed if appropriate and analyzed with one-way ANOVA followed by Student–Newman–Keuls post hoc test and then presented as means with 95% confidence interval. Non-normally distributed or non-homogeneous data were evaluated using Kruskal–Wallis *H* test followed by Conover post hoc test and presented as medians with 95% confidence interval. The *p* values ≤ 0.05 were considered statistically significant. Two-group comparisons were conducted using *t*-test for independent samples with Welch correction in case of unequal variances. Spearman rank correlation was used to analyze the interrelationship between gene expression. All calculated probabilities were two-tailed. Statistical analysis was conducted using MedCalc^®^ Statistical Software version 20 (MedCalc Software Ltd., Ostend, Belgium; https://www.medcalc.org).

## 3. Results

### 3.1. Transcriptomic Analysis

**Figure 1:**
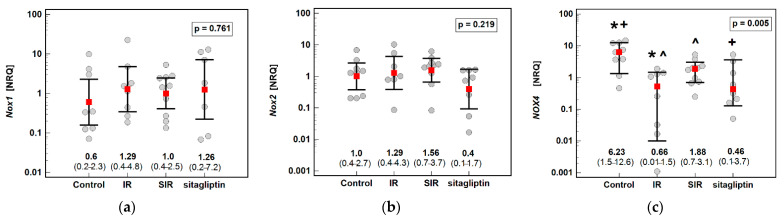
Effect of ischemia/reperfusion injury and sitagliptin on hepatic NADPH oxidase (Nox) expression: (**a**) *Nox1*; (**b**) *Nox2*; (**c**) *Nox4*. Data are presented as geometric means (*Nox1* and *Nox2*) or medians (*Nox4*) with 95% confidence intervals and analyzed with, respectively, one-way ANOVA or Kruskal–Wallis *H* test. Significant (*p* < 0.05) between-group differences are marked by the same type of symbols: *, +, ^. IR, ischemia/reperfusion group; SIR, ischemia/reperfusion group pretreated with sitagliptin; NRQ, normalized relative quantity.

The potential impact of IR injury and pretreatment with sitagliptin on the hepatic expression of NOX enzymes and selected inflammation-related mediators was evaluated. The IR injury significantly downregulated *Nox4* (by 9.4-fold) as compared to the control animals. The sitagliptin pretreatment upregulated *Nox4* expression by 2.9-fold as compared to the animals subjected to the IR procedure alone but did not fully restore the enzyme as *Nox4* expression in the SIR group remained downregulated by 3.3-fold as compared to the control animals (Figure 1).

**Figure 2:**
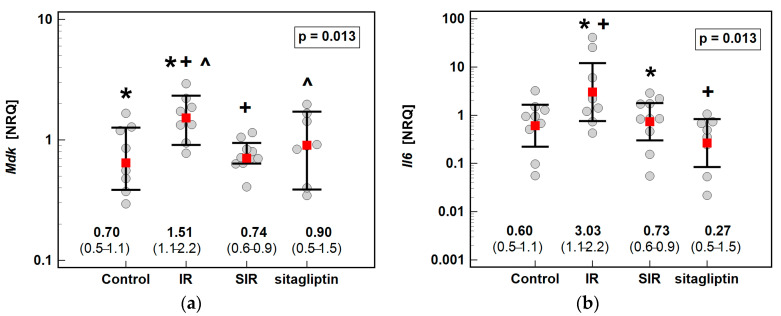
Effect of ischemia/reperfusion injury and sitagliptin on hepatic expression of (**a**) midkine (*Mdk*); (**b**) interleukin-6 (*Il6*). Data are presented as geometric means with 95% confidence intervals and analyzed with one-way ANOVA. Significant (*p* < 0.05) between-group differences are marked by the same type of symbols: *, +, ^. IR, ischemia/reperfusion group; SIR, ischemia/reperfusion group pretreated with sitagliptin; NRQ, normalized relative quantity.

Of the evaluated inflammatory mediators, IR injury and/or sitagliptin had significant effect on the expression of *Mdk* and *Il6* (Figure 2) but not on that of *Mmp1*, *Nampt*, *Ptn* or *Tnfa* (Table 2). Regarding midkine, IR injury caused a significant, 2.2-fold elevation in its expression, which was restored to control animal level in rats pretreated with sitagliptin (Figure 2a). Likewise, sitagliptin prevented *Il6* elevation as cytokine expression in the SIR group was lower by 4.2-fold as compared to the animals subjected to IR alone (Figure 2b).

The expression of *Nampt* and *Ptn* was strongly and positively correlated with each other and moderately with *Nox2* and *Nox4* expression. Of the other analyzed genes, *Nox1* strongly and positively correlated with *Mmp1* and moderately with *Mdk* and *Tnf*, while negatively with *Nox2* and *Nampt*. *Nox4* moderately and inversely correlated with *Nox1*, *Mdk* and *Tnfa* and weakly positively with *Nox2*. Moreover, there were weaker positive correlations between *Il6* and *Nampt* and between *Mdk* and *Mmp1* (Table 3).

### 3.2. Cytoprofiling

**Figure 3:**
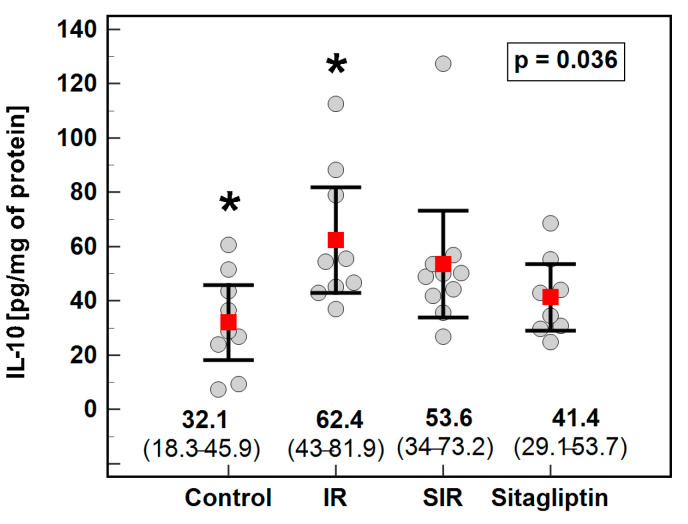
Effect of ischemia/reperfusion injury and sitagliptin on hepatic concentration of IL-10. Data are presented as means with 95% confidence interval (CI), analyzed with one-way ANOVA. Significant (*p* < 0.05) between-group difference is marked by *. IR, ischemia/reperfusion group; SIR, ischemia/reperfusion group pretreated with sitagliptin.

Tissue homogenates were used to determine the potential impact of IR injury with and without pretreatment with sitagliptin on the liver concentration of the selected cytokines and growth factors, namely, IL-1β, IL-6, IL-10, IL-12(p70), IL-13, IFN-γ, MIP-2, TNF-α and VEGF-A. Of those, the concentrations of IL-6, IL-12(p70) and IL-13 were below the limit of detection of the assay in the majority of cases and, thus, were excluded from the analysis. Except for the elevation of IL-10 by two-fold in the IR group as compared to the controls (Figure 3), none of the remaining cytokines differed significantly between the analyzed groups (Table 4).

### 3.3. 3-Nitro- (NT), 3-Chloro- (CT) and 3-Bromotyrosine (BT)

**Figure 4:**
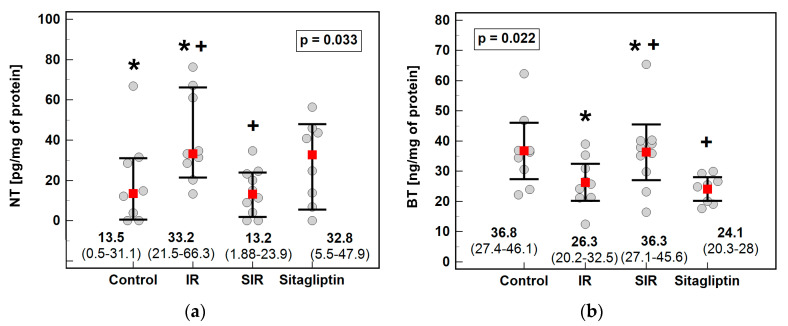
Effect of ischemia/reperfusion injury and sitagliptin on hepatic concentration of (**a**) 3-nitrotyrosine (NT); (**b**) 3-bromotyrosine (BT). Data are presented as medians or means with 95% confidence interval (CI) and analyzed with Kruskal–Wallis *H* test or one-way ANOVA for, respectively, NT and BT. Significant (*p* < 0.05) between-group differences are marked by the same type of symbols: *, +. IR, ischemia/reperfusion group; SIR, ischemia/reperfusion group pretreated with sitagliptin.

The effects of IR injury and sitagliptin on the markers of nitrative and halogenative stress were examined using metabolomic analysis of NT, CT and BT. With the method applied, CT concentrations in liver homogenates were undetectable. The concentration of NT was higher upon IR injury (by 2.5-fold) and at the level comparable to the controls if IR was preceded by sitagliptin treatment (Figure 4a). On the contrary, the BT concentration was lower in the IR injured liver by 1.4-fold and restored to the control level in the animals pretreated with sitagliptin (Figure 4b).

## 4. Discussion

Dipeptidyl peptidase-4 inhibitors are among the drugs studied for their potential use for new therapeutic purposes, a strategy referred to as drug repurposing or repositioning [9]. Recently, a large body of evidence has accumulated showing sitagliptin effectiveness in lessening tissue damage during intestinal [24,32], cardiac [23,33], cerebral [30], testicular [31] and renal [28,29] ischemia-reperfusion insult. Likewise, sitagliptin has reduced the histopathological signs of injury and decreased the serum activities of ALT and AST in animal models of hepatic IR [21,22,25,26], acting via mechanisms employing its anti-inflammatory and antioxidative properties. Accordingly, an alleviation of oxidative stress in the ischemic liver has previously been shown, manifested by upregulated activity and/or the expression of enzymatic antioxidants, including paraoxonase-1 [22], superoxide dismutase (SOD) [22,25] and heme oxygenase [25], which has been accompanied by an increased concentration of reduced glutathione [25] and a diminished level of lipid peroxidation [22,25,26]. Mechanistically, sitagliptin has been demonstrated to upregulate the expression of *Nrf2* [36]. Nrf2 is a key transcription factor involved in stress response to oxidative insult and controlling the expression of a plethora of antioxidants [37]. Adding to previous observations regarding antioxidants and lipid peroxidation markers, we sought a sitagliptin effect on NOX enzymes, a family of NADPH oxidases and one of major ROS-generating systems. Of the seven known mammal forms, NOX1, NOX2 and NOX4 are dominant in the liver [38]. They are expressed by hepatic stellate cells and hepatocytes, while Kupffer cells express NOX2, epithelial cells NOX1 and NOX4, and vascular smooth muscle cells—NOX4 [39]. The NOX2 is also a dominant form of phagocytic cells [39]. Under the experimental conditions of the current study, *Nox1* was not affected. Likewise, no significant changes were observed regarding *Nox2*, although a ten times higher dose of sitagliptin has been shown to downregulate its expression in the fibrotic livers of mice with nonalcoholic steatohepatitis [40]. Upregulated NOX1 and NOX2 expression is considered to contribute to tissue damage and is consistently associated with poor prognosis in patients with hepatocellular carcinoma [41]. NOX4, in turn, is credited with hepatoprotective properties and its high expression is linked with a better prognosis [41]. Herein, *Nox4* expression was modulated by sitagliptin, and the effect reached statistical significance, despite a relatively low drug dose. Pretreatment with the drug partially restored *Nox4* expression, which was markedly (by 9.4-fold) downregulated by the IR procedure. Again, the *Nox4*-reinstating properties of sitagliptin manifested themselves solely under ischemic conditions. The cytoprotective nature of NOX4 has been observed in the cardiovascular system, where its upregulated expression enhanced vasodilation, decreased blood pressure and reduced IR-induced infarct size. The effect has been attributed to NOX4′s ability to generate H_2_O_2_ instead of superoxide anion, which may prevent superoxide-mediated NO inactivation (reviewed in [42]).

In addition to inducing the expression of antioxidants, Nrf2 negatively affects inflammation by directly blocking the expression of IL-1β, IL-6 and NFκB activity [37]. In fact, gliptins have been claimed to be more powerful anti-inflammatory agents than antioxidants [40]. Consistently, *Il6* upregulation in ischemic liver, but not in IR animals pretreated with sitagliptin, was observed in the current study, corroborating previous findings [26]. Sitagliptin has reportedly similar lowering properties on TNFα, as shown in ischemic liver [26] or in animals with induced hepatotoxicity by treatment with methotrexate [43] or acetaminophen [36]. Still, we did not observe any effect of either IR or sitagliptin on TNFα, on mRNA or the protein level. The discrepancy may be explained by a lower drug those by two-to-sixty-fold as compared to other studies on liver toxicity or IR [23,30,31,36,43]. Likewise, neither IR nor sitagliptin affected the concentrations of IL-1β, IFN-γ, MIP-2 or VEGF-A, or the expression of *Mmp1*, although their upregulation upon IR insult and/or downregulation by sitagliptin might be expected [43,44,45], or vice versa in the case of VEGF-A [46].

In addition, we investigated sitagliptin’s effect on the expression of other mediators of inflammation, namely, nicotinamide phosphoribosyltransferase/visfatin (*Nampt*), pleiotrophin (*Ptn*) and midkine (*Mdk*). The rationale for seeking Nampt’s association with IR and sitagliptin was a recent finding on a crosstalk between the adipose tissue and the liver during hepatic IR [47]. Moreover, Nampt has been shown to promote mitochondrial integrity and ensure cell survival upon oxidative challenge by activating Nrf2 and its downstream signaling [48]. Furthermore, sitagliptin, by inhibiting DPP-4 and, thus, preserving GLP-1, may influence Nampt/visfatin production. GLP-1 has been shown to downregulate the Nampt/visfatin expression and its release from adipocytes [49]. Intriguingly, GLP-1 has been speculated to have a similar effect on leukocytes [50]. However, the low dose of sitagliptin used in the current study had no significant effect on *Nampt*.

Midkine and pleiotrophin are the only members of the multifunctional cytokine/heparin-binding growth factor family [51]. Similar to *Nampt*, *Ptn* expression was not significantly affected, although it has been upregulated in microglia following IR injury, where it played a neuroprotective role [52]. Likewise, pleiotrophin has displayed angiogenic properties and the stimulated formation of new blood vessels in a cardiac model of ischemia [53]. In turn, the cytoprotective properties of midkine, associated with its pro-survival activity, have been demonstrated in cardiac IR injury model [54]. Still, in line with midkine’s role as a potent chemoattractant for neutrophils and macrophages (reviewed in [51]), midkine has been shown to contribute to tissue damage in renal [55] and hindlimb ischemia [56]. Contrary to *Ptn*, but corroborating the aforementioned findings, *Mdk* was significantly upregulated following IR insult. Moreover, sitagliptin reduced its expression, both in the sham-operated and the IR animals. Supporting a possibly negative role in hepatic IR, midkine was positively correlated with *Nox1* and *Mmp1* and negatively with *Nox4*. There are no data regarding midkine and pleiotrophin in hepatic IR injury. Nonetheless, they have been shown to participate in both inflammatory and regenerative processes after partial hepatectomy and their net role was concluded to be beneficial [57]. In addition, midkine overexpression in the liver of animals exposed to cadmium has been claimed to protect against metal-induced toxicity by reducing apoptotic rates [58]. On the other hand, however, midkine is a powerful chemoattractant for neutrophils [51], which are of particular importance in the pathogenesis of liver diseases [59]. Only recently, the formation of a neutrophil extracellular trap (NET) has been linked to hepatic IR. It has been shown that NOX-generated ROS activate peptidyl arginine deiminase IV (PAD4), which starts chromatin decondensation and NET formation [60]. For this reason, it is of particular interest that midkine has been found to be involved in promoting NETosis in myocarditis [61].

The IR injury was accompanied by, rather unexpected, a significant elevation of IL-10. Still, as already mentioned, hepatic IR is associated with NET formation [60]. In turn, NET removal by macrophages is accompanied by IL-10 release in addition to IL-6 [62], potentially explaining the upregulation of both IL-10 and *Il6* observed in our study.

IR injury is associated with an upregulated expression of NOS2 [63,64], an inducible form of nitric oxide synthase, which contributes to RNS generation. It is generally believed to result in nitrative stress. However, some beneficial aspects of NOS2 upregulation are reported as well (reviewed in [65]). Sitagliptin has been shown to reduce NOS2 expression [25,43] and decrease NOS2-generated NO [25], while inducing the expression of the endothelial isoform of the enzyme (NOS3) [23]. Leukocyte- and intracellular-generated RNS lead to the formation of nitro-adducts in ischemic hepatocytes [4]. Accordingly, immunoreactivity toward total NT (mostly protein-bound form) has been shown to increase upon IR injury, more so at 2 than 24 h [66]. We showed that free NT, quantified here using a mass spectrometry-based method, was also increased in ischemic liver. Moreover, sitagliptin pretreatment prevented its elevation under IR conditions, although there was no effect in the sham-operated animals. This is partly in line with our previous observations regarding the effect of this drug on selected parameters of the NO-ADMA-DDAH pathway. The 14-day treatment with sitagliptin resulted in an increase in the L-arginine/ADMA ratio in the non-ischemic group and an increase in the activity of dimethylarginine dimethylaminohydrolase (DDAH) in the ischemic group [21]. The l-Arginine/ADMA ratio reflects l-arginine bioavailability for NOS, while DDAH is an enzyme metabolizing ADMA, the main NOS inhibitor [67].

Ischemia also upregulates myeloperoxidase (MPO) activity, while sitagliptin causes its downregulation [30]. MPO uses halides (Cl^−^, Br^−^, I^−^) to form highly reactive hypohalites, with HOCl having the highest oxidative ability, followed by HOBr [5]. An accelerated generation of RHS causes halogenative stress and leads to the formation of halogen-adducts in IR-stressed hepatocytes [4]. HOCl synthesis by MPO exceeds that of HOBr owing to the higher availability of Cl^−^. Therefore, CT is considered a neutrophil activation marker [5]. In turn, eosinophil peroxidase preferentially generates HOBr and, therefore, BT is regarded as an eosinophil activation marker [6]. To the best of our knowledge, data on halogenative stress in hepatic IR injury are scarce. The targeted metabolomic method applied here allowed us to quantify free BT in liver homogenates but not CT. Higher concentrations of free BT than CT might result from liver infiltration with eosinophils, as recently demonstrated in human liver transplants [68], and from the fact that, depending on the pH, neutrophil MPO may generate HOBr as a dominant oxidant [69]. Still, the BT concentrations were low, tittering at the verge of the method’s limit of detection or dropped below when water-based homogenates, favoring NT quantification, were used. Therefore, we reassessed BT using homogenates prepared with a lysis buffer containing deoxycholate, which, owing to the higher predicted pKa value for BT than NT, improved metabolite recovery. Even more importantly, the slightly alkaline pH of the lysis buffer markedly improved BT solubility. Unlike NT, IR injury was accompanied by lower free BT concentration and sitagliptin prevented its drop under IR conditions without a significant effect in the sham-operated animals. The pattern displayed by BT resembled that of cytoprotective *Nox4*. Correspondingly, a hepatoprotective role during IR injury has been attributed to eosinophils [68]. Moreover, dibromotyrosine displayed neuroprotective properties in in vitro models of brain ischemia [70]. Sitagliptin, in turn, has been shown to upregulate eotaxin-1, a major chemoattractant for eosinophils [71]. However, iodotyrosine dehalogenase 1 (DEHAL1) presence has been detected in the liver [72]. If upregulated during IR injury, it might be associated with accelerated BT dehalogenation [72]. In this respect, it would be of interest to determine IR’s effect on DEHAL1. To further address the issue of a possible accelerated BT dehalogenation, free BT quantification ought to be combined with a determination of 4-hydroxyphenylacetic acid (HPA), its degradation product, in urine.

The major limitation of our study is the relatively low dose of sitagliptin, which is likely to contribute to a lack of statistical significance of some trends observed here. However, it is a follow-up study, and the experiments were conducted based on the sitagliptin doses used in early research on its role in IR injury [18,28], which were markedly lower than those used in more recent studies. Nonetheless, it only strengthens the relevance of observations found to be statistically significant. Another limitation that ought to be mentioned is the lack of measurements of myeloperoxidase and eosinophil peroxidase activities as well as a lack of data on tissue infiltration with eosinophils, which would complement and help the interpretation of the results regarding halogenative stress.

## 5. Conclusions

Here, we demonstrated that an IR procedure upregulates the expression of chemoattractant midkine and increases the concentration of free 3-nitrotyrosine, a nitrative stress marker and IL-10, while downregulating the expression of cytoprotective *Nox4* in ischemic livers. We also showed that even a low sitagliptin dose can alleviate IR injury as it partly restores *Nox4* expression, decreases the expression of inflammatory mediators, interleukin 6 and midkine, and reduces the concentration of free 3-nitrotyrosine, while increasing that of 3-bromotyrosine. Therefore, the anti-inflammatory and antioxidant properties of sitagliptin had been confirmed and the drug’s ability to modulate nitrative and halogenative stress during hepatic IR injury have been demonstrated.

## Figures and Tables

**Table 1:** Primers’ sequences.

Gene	Forward Sequence	Reverse Sequence
*Gapdh*	TGACTCTACCCACGGCAAGTTCAA	ACGACATACTCAGCACCAGCATCA
*Il6*	ACAGCGATGATGCACTGTCAG	ATGGTCTTGGTCCTTAGCCAC
*Tnfa*	GCCCAGACCCTCACACTC	CCACTCCAGCTGCTCCTCT
*Mmp1*	CCACTAACATTCGAAAGGGTTT	GGTCCATCAAATGGGTTATTG
*Nampt*	TCTGGAAATCCGCTCGACAC	TATCCACTCCGTCCCCTTGA
*Mdk*	TGGAGCCGACTGCAAATAC	TGTACCGAGCCTTCTTCAGG
*Nox1*	TTCCCTGGAACAAGAGATGG	GACGTCAGTGGCTCTGTCAA
*Nox2*	CTGCCAGTGTGTCGGAATCT	TGTGAATGGCCGTGTGAAGT
*Nox4*	GGATCACAGAAGGTCCCTAGC	AGAAGTTCAGGGCGTTCACC
*Ptn*	TGGAGCTGAGTGCAAATAC	TGTGCAGAGCTCTCTTCAGA

*Gapdh*, glyceraldehyde 3-phosphate dehydrogenase; *Il6*, interleukin 6; *Tnfa*; tumor necrosis factor α; *Mmp1*, matrix metalloproteinase-1; *Nampt*, nicotinamide phosphoribosyltransferase; *Mdk*, midkine; *Nox*, NADPH oxidase; *Ptn*, pleiotrophin.

**Table 2:** Effect of ischemia/reperfusion (IR) injury and/or sitagliptin on hepatic expression level of selected inflammatory mediators.

Gene	Mean (95% CI) (NRQ)				*p*
	Control	IR	SIR	Sitagliptin	
*Mmp1*	0.37 (0.1–1.0)	1.09 (0.1–9.4)	0.68 (0.2–2.9)	3.36 (0.3–38.7)	0.244
*Nampt*	1.12 (0.5–2.5)	1.25 (0.5–3.0)	1.39 (0.8–2.4)	0.50 (0.2–1.3)	0.158
*Ptn*	1.25 (0.7–2.4)	1.0 (0.6–1.6)	1.21 (0.8–1.8)	0.66 (0.4–1.1)	0.172
*Tnfa*	0.77 (0.4–1.5)	1.07 (0.3–4.2)	1.21 (0.6–2.6)	0.81 (0.3–2.6)	0.839

Gene expression expressed as normalized relative quantities (NRQ) and presented as geometric means with 95% confidence interval (CI), analyzed with one-way ANOVA. IR, ischemia/reperfusion group; SIR, ischemia/reperfusion group pretreated with sitagliptin; *Mmp1*, matrix metalloproteinase-1; *Nampt*, nicotinamide phosphoribosyltransferase; *Ptn*, pleiotrophin; *Tnfa*; tumor necrosis factor α.

**Table 3:** Interrelationships between expression of analyzed inflammation-related genes.

Gene	*Nampt*	*Mmp1*	*Nox2*	*Il6*	*Nox1*	*Mdk*	*Tnfa*	*Nox4*
*Ptn*	0.76 ^3^	ns	0.52 ^2^	ns	ns	ns	ns	0.47 ^2^
*Nampt*		ns	0.60 ^3^	0.34 ^1^	−0.38 ^1^	ns	−0.41 ^1^	0.45 ^2^
*Mmp1*			−0.41 ^1^	ns	0.73 ^3^	0.41 ^1^	ns	ns
*Nox2*				ns	−0.43 ^2^	ns	ns	0.35 ^1^
*Il6*					ns	ns	ns	ns
*Nox1*						0.49 ^2^	0.53 ^3^	−0.56 ^3^
*Mdk*							ns	−0.49 ^2^
*Tnfa*								−0.44 ^2^

Data presented as Spearman correlation coefficients (ρ). ^1^ *p* ≤ 0.05; ^2^ *p* ≤ 0.01; ^3^ *p* ≤ 0.001; ns, non-significant (*p* > 0.05). Magnitude of relative change is shown in the form of a four-color-scaled heatmap (red-yellow-green-blue, scaled from −1.0 (dark blue) to 1.0 (dark red).

**Table 4:** Effect of ischemia/reperfusion (IR) injury and/or sitagliptin on hepatic concentration of selected cytokines and growth factors.

Cytokine	Mean (95% CI) (pg/mg of Protein)				*p*
	Control	IR	SIR	Sitagliptin	
IL-1β	46.7 (27.3–66.1)	48.6 (23.2–74)	50.8 (44.3–57.2)	62.1 (37.4–86.8)	0.613
IFN-γ	211.8 (123–300)	201.5 (136–267)	207.1 (138–276)	209.7 (173–246)	0.995
MIP-2	104.6 (63–146)	91.4 (75–108)	103 (77–129)	90.7 (51–130)	0.825
TNF-α	0.90 (0.2–1.6)	0.49 (0.2–0.8)	0.73 (0.2–1.3)	0.57 (0.4–0.8)	0.551
VEGF-A	31.7 (22.9–40.5)	34.6 (26.9–42.4)	39.0 (32.1–46)	37.3 (30.2–44.4)	0.426

Data are presented as means with 95% confidence interval (CI), analyzed with one-way ANOVA. IR, ischemia/reperfusion group; SIR, ischemia/reperfusion group pretreated with sitagliptin; IL, interleukin; IFN, interferon; MIP, macrophage inflammatory protein; TNF, tumor necrosis factor; VEGF, vascular endothelial growth factor.

## Data Availability

Data are included in the article.
